# Genomic dynamics of species and mobile genetic elements in a prolonged *bla*_IMP-4_-associated carbapenemase outbreak in an Australian hospital

**DOI:** 10.1093/jac/dkz526

**Published:** 2020-01-20

**Authors:** A Kizny Gordon, H T T Phan, S I Lipworth, E Cheong, T Gottlieb, S George, T E A Peto, A J Mathers, A S Walker, D W Crook, N Stoesser

**Affiliations:** 1 Nuffield Department of Medicine, University of Oxford, Oxford, UK; 2 NIHR Health Protection Research Unit in Healthcare Associated Infections and Antimicrobial Resistance at University of Oxford in partnership with Public Health England, Oxford, UK; 3 Department of Microbiology & Infectious Diseases, Concord Repatriation General Hospital, Sydney, Australia; 4 University of Sydney, Sydney, Australia; 5 NIHR Oxford Biomedical Research Centre, University of Oxford/Oxford University Hospitals NHS Foundation Trust, Oxford, UK; 6 Division of Infectious Diseases and International Health, University of Virginia, Charlottesville, VA, USA

## Abstract

**Background:**

Hospital outbreaks of carbapenemase-producing organisms, such as *bla*_IMP-4_-containing organisms, are an increasing threat to patient safety.

**Objectives:**

To investigate the genomic dynamics of a 10 year (2006–15) outbreak of *bla*_IMP-4_-containing organisms in a burns unit in a hospital in Sydney, Australia.

**Methods:**

All carbapenem-non-susceptible or MDR clinical isolates (2006–15) and a random selection of equivalent or ESBL-producing environmental isolates (2012–15) were sequenced [short-read (Illumina), long-read (Oxford Nanopore Technology)]. Sequence data were used to assess genetic relatedness of isolates (Mash; mapping and recombination-adjusted phylogenies), perform *in silico* typing (MLST, resistance genes and plasmid replicons) and reconstruct a subset of *bla*_IMP_ plasmids for comparative plasmid genomics.

**Results:**

A total of 46/58 clinical and 67/96 environmental isolates contained *bla*_IMP-4_. All *bla*_IMP-4_-positive organisms contained five or more other resistance genes. *Enterobacter cloacae* was the predominant organism, with 12 other species mainly found in either the environment or patients, some persisting despite several cleaning methods. On phylogenetic analysis there were three genetic clusters of *E. cloacae* containing both clinical and environmental isolates, and an additional four clusters restricted to either reservoir. *bla*_IMP-4_ was mostly found as part of a cassette array (*bla_IMP-4_-qacG2-aacA4-catB3*) in a class 1 integron within a previously described IncM2 plasmid (pEl1573), with almost complete conservation of this cassette across the species over the 10 years. Several other plasmids were also implicated, including an IncF plasmid backbone not previously widely described in association with *bla*_IMP-4._

**Conclusions:**

Genetic backgrounds disseminating *bla*_IMP-4_ can persist, diversify and evolve amongst both human and environmental reservoirs during a prolonged outbreak despite intensive prevention efforts.

## Introduction

Carbapenems are agents of last resort to treat MDR Gram-negative bacterial infections. Of concern, the global incidence of infections with carbapenem-resistant organisms (CROs) is increasing, and mortality rates of up to 50% are reported.^1^ The production of carbapenemases, such as *Klebsiella pneumoniae* carbapenemase (KPC), oxacillinase-48 (OXA-48) and the MBLs, is the most problematic mechanism of carbapenem resistance as genes encoding these enzymes are readily transferred between organisms on mobile genetic elements (MGEs) such as plasmids.

Imipenemases (encoded by *bla*_IMP_ variants) are MBLs that were first identified on MGEs in *Pseudomonas aeruginosa* in Japan in the late 1980s.^2^ Since then, there has been worldwide detection of *bla*_IMP_ alleles in non-glucose-fermenting Gram-negative rods (mostly *P. aeruginosa* and *Acinetobacter* spp.) and Enterobacterales, although they are predominantly found in Asia.^3^ Most *bla*_IMP_ variants have been identified in distinct geographical regions; however, some (e.g. *bla*_IMP-1_, *bla*_IMP-4_ and *bla*_IMP-7_) are more widely disseminated, showing their potential for intercontinental spread.^4^*bla*_IMP_ is generally embedded in class 1 (predominantly) and class 3 integrons flanked by transposons within plasmids, which facilitate its horizontal dissemination.^3^^,^^5^ Additionally, *bla*_IMP_ often co-exists with other resistance genes, causing clinically problematic cross-class resistance.

*bla*_IMP-4_ was first identified in the mid-1990s in *Acinetobacter* spp. in Hong Kong and in *Citrobacter youngae* in China.^6^^,^^7^ By 2004 there were simultaneous reports of *bla*_IMP-4_ in *P.* *aeruginosa* and Enterobacterales in hospitals in Sydney and Melbourne,^8^^,^^9^ and it is currently the predominant carbapenemase gene detected in Australia. More recently it has also been described in the USA,^10^ France^11^ and other parts of Asia.^12^^,^^13^ Hospital-associated outbreaks with IMP-4-positive organisms have been reported in Australia and China.^14–18^

*bla*_IMP-4_ has been described in at least seven different broad host range Inc group plasmid types (A/C, A/C-Y, HI2, HI2-N, I1, L/M and N1) over the last 15 years^19^^,^^20^ and, although it is always found in class 1 integrons, it may be found as a single gene cassette or as part of a cassette array.^5^^,^^19^^,^^21^ Most commonly it is present in a four-gene cassette array: *bla*_IMP-4_-*qacG-aacA4-catB3*, found across Australia,^16^^,^^19^^,^^21^^,^^22^ Hong Kong,^17^^,^^21^^,^^23^ Singapore,^12^^,^^21^ Japan^5^ and Malaysia.^24^ This array also contains genes conferring resistance to quaternary ammonium compounds (*qacG*), some aminoglycosides (*aacA4*) and chloramphenicol (*catB3*).

A burns unit in Sydney, Australia, experienced prolonged *bla*_IMP-4_ transmission in patients from 2006 to 2015, with environmental contamination with IMP-4-positive organisms noted in 2008.^14^ There are limited epidemiological data available on large, prolonged IMP-4 outbreaks. We used WGS [Illumina (all isolates) and Oxford Nanopore Technology (ONT) (*n *=* *20)] to identify genetic modes of *bla*_IMP-4_ transmission and overlap between patient and environmental reservoirs, and to study the evolutionary dynamics of *bla*_IMP-__4_-associated MGEs in a single centre over 10 years.

## Materials and methods

### Sampling, microbiology, antimicrobial susceptibility testing and detection of carbapenemases

All carbapenem-non-susceptible, *bla*_IMP-4_-positive or MDR (resistant to three or more classes of antimicrobials) clinical isolates from Concord Repatriation General Hospital (CRGH), Sydney, Australia, from 2006 to 2015 were collected and stored. A random selection of carbapenem-non-susceptible, *bla*_IMP-4_-positive, MDR- or ESBL-producing isolates from the environment of the burns unit at CRGH collected from 2012 to 2015 were stored. Methods of specimen collection, organism detection, antibiotic susceptibility testing and carbapenemase PCR have been previously reported.^14^ For this study, all stored organisms were re-cultured on Columbia Blood Agar (CBA) (Thermo Fisher Scientific Oxoid, Basingstoke, UK) to check for viability and purity. A 10 μg ertapenem disc was used to confirm carbapenem non-susceptibility. Mixed cultures were subcultured separately from single colony picks, and species were identified using MALDI-TOF to determine if they were true mixed cultures or within-species culture variation. Approximately 10 colonies from pure culture were stored as a single stock in nutrient broth with glycerol at −80°C.

### WGS

Stock cultures were subcultured on CBA with a 10 μg ertapenem disc. Approximately five colonies closest to the ertapenem disc were selected for DNA extraction. For short-read sequencing using the Illumina HiSeq 2500, DNA extraction was performed using the QuickGene DNA extraction kit (Autogen, Holliston, MA, USA) as per the manufacturer’s instruction, plus an additional mechanical lysis step following chemical lysis (FastPrep, MP Biomedicals, CA, USA; 6 m/s for 40 s). For long-read sequencing using ONT MinION, DNA was extracted using the Qiagen Genomic-tip 100/G kit (Qiagen, Hilden, Germany). DNA quantity and fragment size distributions were assessed using Qubit fluorometry (Thermo Fisher Scientific, Waltham, MA, USA) and the TapeStation system (Agilent, Santa Clara, CA, USA), respectively. DNA was fragmented to ∼25 kb using Covaris g-TUBE (Covaris, Inc., Woburn, MA, USA) and normalized to 4 μg. Sequencing libraries were prepared following the manufacturer’s protocol (ONT, Oxford, UK) and using the Ligation Sequencing Kit 1 D (SQK-LSK108) with the Native Barcoding Kit 1 D (EXP-NBD103). Each sequencing library consisted of four multiplexed samples loaded onto R9.4 Flow Cells (FLO-MIN106). Sequencing was performed on the MinION device controlled by MinKNOW software (v.1.7.10–v.1.7.7); the raw data were base called by Albacore (v.1.1.0–v.1.2.5).

### Sequence data processing and analyses

Short-read sequences were initially processed using a previously described pipeline^25^ incorporating read trimming/quality control, species classification (Kraken^26^), read mapping to species-associated reference genomes, variant calling and filtering, and the production of consensus fasta sequences of variant calls with respect to the selected reference. Species-specific references used included: *Enterobacter cloacae* (GenBank accession: CP001918.1), *Escherichia coli* (AE014075.1), *K. pneumoniae* (CP000647.1), *Klebsiella oxytoca* (NC_018106.1) and *Serratia marcescens* (NC_020211.1). For these species, recombination-adjusted phylogenies were created from core chromosomal single nucleotide variants (SNVs) (padded to the length of the reference genome prior to analysis) using IQtree (flags: -m GTR+G -blmin 0.00000001 -t PARS)^27^ and then ClonalFrameML^28^ (default parameters).

We used SPAdes^29^ v3.6 to *de novo* assemble the isolates from raw reads (default options/parameters). BLASTn comparisons of assemblies with species-appropriate seven locus MLST databases (https://pubmlst.org/databases/) were used for MLST typing, requiring 100% matches to call alleles. Resistance gene, insertion sequence and plasmid typing were performed using resistType (https://github.com/hangphan/resistType; also a BLASTn-based method), and the ResFinder,^30^ PlasmidFinder^31^ and ISFinder^32^ databases, respectively.

A pan-species phylogeny was created using Mashtree^33^ v0.57 (default parameters: kmer length = 21, sketch size = 10 000), with sketches created from the SPAdes *de novo* assemblies. For *Citrobacter* *freundii* and *Leclercia* *adecarboxylata*, we used Mash distances from this phylogeny as proxy measures of genetic relatedness.

2D reads were extracted from ONT MinION sequence data using poretools,^34^ and Unicycler^35^ was used to generate hybrid assemblies from MinION and Illumina data (default options/parameters). Sequences were annotated using PROKKA,^36^ with some manual modifications.

Sequence data have been deposited in GenBank (BioProject accession: PRJNA550014).

### Data visualization

Data were visualized using FigTree v1.4.3 (http://tree.bio.ed.ac.uk/software/figtree/); R (version 3.43);^37^ ggtree version 1.16.0 and ggplot2; Geneious (v9.0.4; https://www.geneious.com); and Microreact (https://microreact.org/showcase).

### Ethics

Ethics approval for this study was granted by the Sydney Local Health District Human Research Ethics Committee (ref. CH62/6/2016-021).

## Results

### Isolates and resistance genes

**Figure 1.**
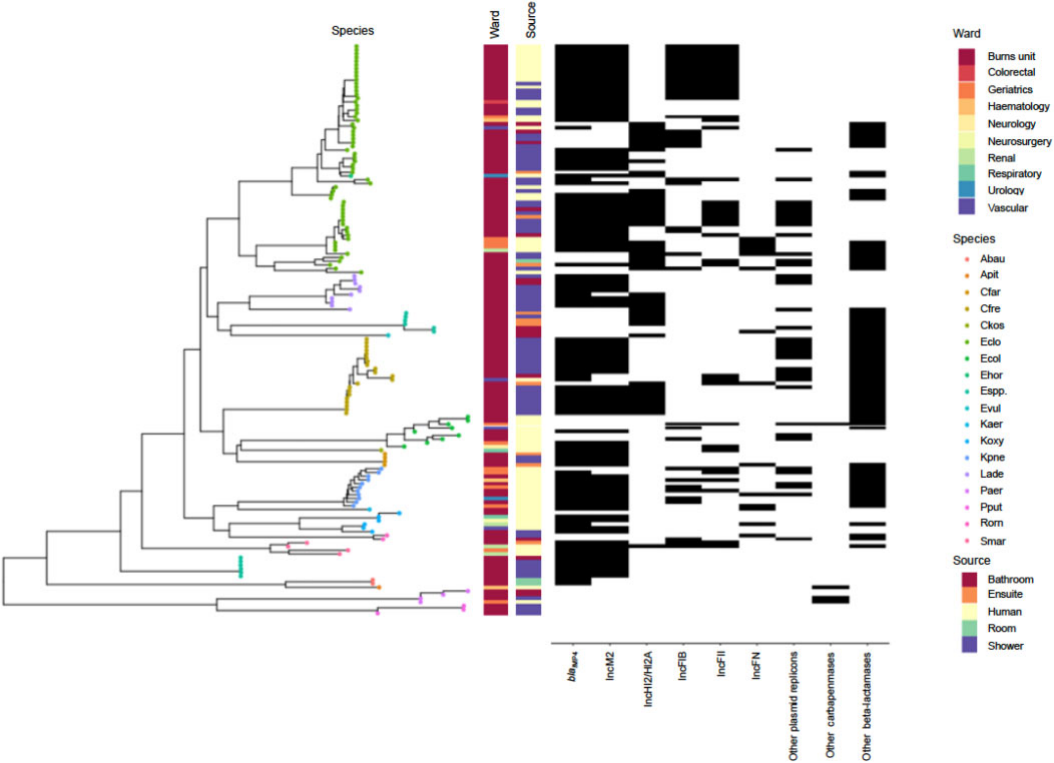
Mash-based phylogeny of all isolates, with species, source/niche, ward, resistance gene and plasmid annotations. Abau, *A. baumanii*; Apit, *A. pittii*; Cfar, *Citrobacter farmeri;* Cfre, *C. freundii*; Ckos, *Citrobacter koseri*; Eclo, *E. cloacae*; Ecol, *E. coli*; Ehor, *Enterobacter hormaechei*; Espp., *Enterobacter* spp.; Evul, *Escherichia vulneris*; Kaer, *Klebsiella aerogenes*; Koxy, *K. oxytoca*; Kpne, *K. pneumoniae*; Lade, *L. adecarboxylata*; Paer, *P. aeruginosa*; Pput, *Pseudomonas putida*; Rorn, *Raoultella ornithinolytica*; Smar, *S. marcescens.* An interactive version of this tree is hosted at: https://microreact.org/project/bThb9f7Yp.

Fifty-eight carbapenem-non-susceptible or MDR Enterobacterales/*Pseudomonas* spp./*Acinetobacter* spp. clinical isolates were collected from the burns unit (*n *=* *28) or other units (*n *=* *30; Figure 1), and included the following samples: wound swabs (*n *=* *21), mid-stream/catheter specimen urine (*n *=* *17), rectal/groin screening swabs (*n *=* *5), blood cultures (*n *=* *4), vascular catheter tips (*n *=* *3), tissue/bone (*n *=* *3), sputum (*n *=* *2), peritoneal dialysis fluid (*n *=* *1), peritoneal swab (*n *=* *1) and faeces (*n *=* *1). Ninety-six carbapenem-non-susceptible, ESBL-producing or MDR isolates from the environment in the burns unit were stored. Environmental isolates were cultured from shower drains (*n *=* *36), sluices (*n *=* *16), taps (*n *=* *3) and equipment (*n *=* *15); bathroom drains (*n *=* *13), sinks (*n *=* *4) and sluices (*n *=* *7); and patient room equipment (*n *=* *2) (Table S1, available as Supplementary data at *JAC* Online).

*E. cloacae* was the predominant clinical (24/58 isolates) and environmental species (37/96 isolates) cultured. However, 18 species were cultured; other major species identified included *C. freundii* (1 clinical and 20 environmental), *K. pneumoniae* (11 clinical) and *L. adecarboxylata* (10 environmental) (Figure 1). These other species were mainly found either in patients (*Klebsiella* spp., *E. coli* and *S. marcescens*) or in the environment (*Citrobacter* spp., *L. adecarboxylata* and *Pseudomonas* spp.) (Figure 1).

Twelve species contained *bla*_IMP-4_ [46/58 (79%) clinical and 67/96 (70%) environmental isolates] (Figure 1), consistent with a highly diverse, poly-species, gene-associated outbreak. Of the *bla*_IMP-4_-positive clinical isolates, 46% were from burns unit patients, suggesting that this was the epicentre of the clinical outbreak, with subsequent dissemination to other hospital locations, and/or new, sporadic, introduction of strains into other areas of the hospital. *bla*_IMP-4_-positive environmental isolates from the burns unit were mostly identified in wet areas [shower and bathroom floor drains (40/67); and sluices (13/67)]. *bla*_IMP-4_-positive organisms were not found in patient sinks in the burns unit.

Three other carbapenemase genes were detected; one clinical *E. coli* (2013) contained *bla*_NDM-5_, one clinical *Acinetobacter pittii* (2014) contained *bla*_IMP-26_ and one *P. aeruginosa* clinical (2006) and environmental (2012) isolate each contained *bla*_GES-5_. All *bla*_IMP-4_-positive isolates also contained *bla*_TEM-1_, and *aac*, *mph*, *catB3* and *sul* genes encoding aminoglycoside acetyltransferases, macrolide phosphotransferases, chloramphenicol acetyltransferase and dihydropteroate synthases, respectively, demonstrating the potential for multi-class resistance. The plasmid-mediated quinolone resistance gene *qnrB2* was also detected in 85% (96/113) of *bla*_IMP-4_-positive organisms. Multiple other antimicrobial resistance genes were detected in some *bla*_IMP-4_-positive organisms (Table S1).

### WGS and phylogenetic analyses

The phylogeny based on Mash distances demonstrated clear clustering of genera and species, as expected, and that *bla*_IMP-4_ was widely distributed amongst these, consistent with widespread horizontal gene transfer (Figure 1).

**Figure 2.**
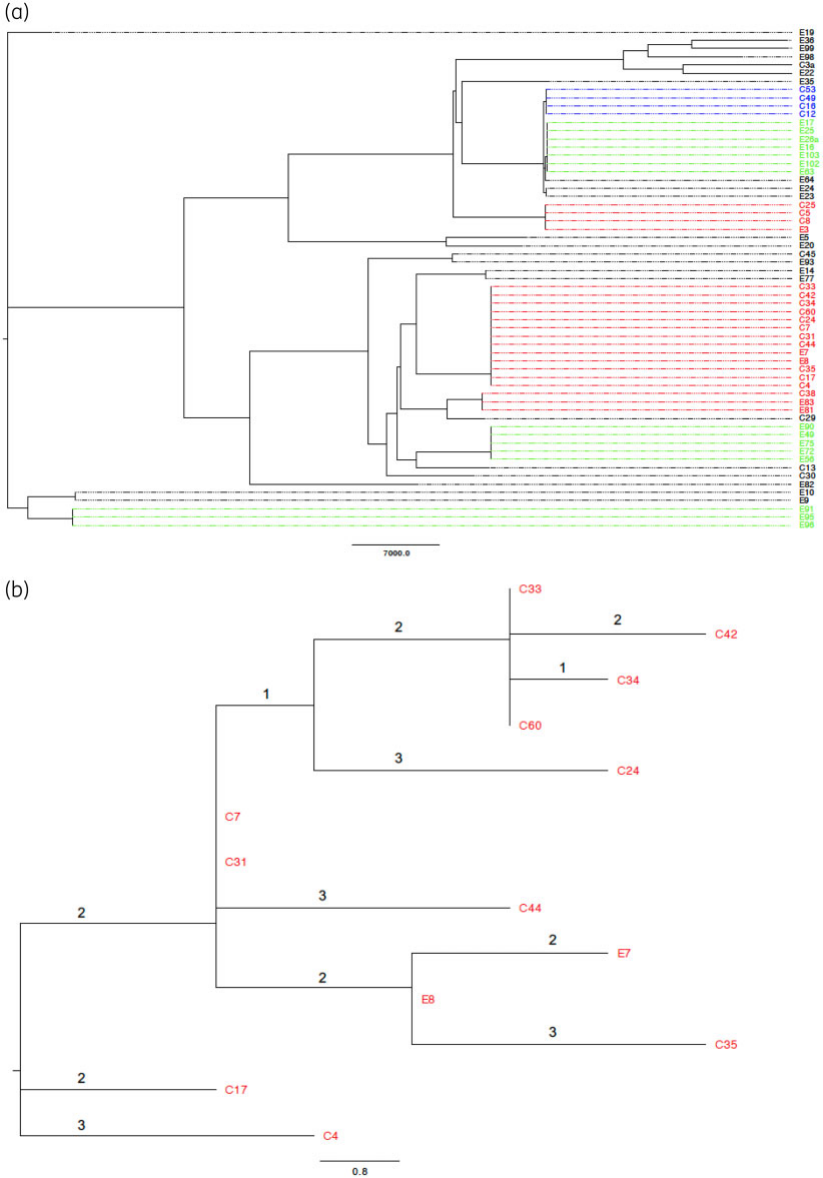
(a) Recombination-adjusted phylogeny of *E. cloacae* isolates using a reference mapping/variant-calling approach and visualized using FigTree. Colours indicate genetic clusters: red, clusters of clinical and environmental isolates; blue, clusters of clinical isolates only; green, clusters of environmental isolates only. Scale represents SNVs. (b) Recombination-adjusted phylogeny of the largest cluster of *E. cloacae* containing both clinical and environmental isolates using a reference mapping/variant-calling approach and visualized using FigTree. Scale represents SNVs.

*E. cloacae* was the predominant organism in both clinical and environment samples. The total diversity across all mapped *Enterobacter* isolates was 138 576 SNVs. Three genetic clusters (three or more isolates, <25 SNVs) of *E. cloacae* contained both clinical and environmental isolates (Figure 2a, red labels). The largest cluster involved 13 isolates (11 clinical and 2 environmental) of ST113, all collected from the burns unit between 2007 and 2012. All 13 contained *bla*_IMP-4_ and were closely genetically related (0–10 SNVs, consistent with published mutation rates of 0.5–3 SNVs/year for *E. cloacae*),^38^ probably representing ongoing transmission between patients and environmental reservoirs over 5 years (Figure 2b). Another cluster of ST269 contained three clinical and one environmental isolate collected from the burns unit between 2007 and 2012. The two earlier clinical isolates (2007–08) were only three SNVs apart, suggesting likely transmission between patients, or from an unidentified environmental reservoir. The latter two isolates from a patient (2010) and the environment (2012) were less closely related (16 SNVs apart), did not contain *bla*_IMP-4_ and differed from the first two isolates by 4–12 SNVs, possibly representing loss of *bla*_IMP-4_, or a new introduction into the unit. The third cluster of ST170 involved three isolates (two environmental and one clinical) collected from the burns unit between 2012 and 2014. They all contained *bla*_IMP-4_, but there were 14 SNVs between the clinical (2012) and the two environmental isolates (both collected on the same day in 2014, from different equipment in the same shower). There were four clusters (three or more isolates) of *E. cloacae* that contained only clinical or environmental isolates (Figure 2a), consistent with small outbreaks of patient-associated cases, and persistence of certain strains in the environment.

A group of 17 environmental *C. freundii* isolates (ST95) was collected from the burns unit between 2012 and 2014, with all but one of these isolates, collected in 2013, harbouring *bla*_IMP-4_. A total of 14/17 were collected from the same shower (drains, sluice and shower trolley) between July 2012 and October 2014, both before (5/14) and after (9/14) environmental cleaning with phenolic agents, chlorine and hydrogen peroxide vapour,^14^^,^^25^ suggesting durability despite physical cleaning and disinfection. The other isolates in this group were collected from drains in another shower (*n *=* *2) and bathroom (*n *=* *1) between 2012 and 2014, illustrating transmission and endurance within the water-based environment of the burns unit. E33 (burns unit bathroom drain; 5 December 2012) and C50 (clinical, vascular surgery; 29 May 2014) were both ST8, with the same distinct resistance genes (including *bla*_IMP-4_, *bla*_VEB-3_, *bla*_OXA-1_ and *bla*_OXA-10_), plasmid Inc types (IncA/C/IncFII) and insertion sequence profiles (Table S1), suggesting the spread of this strain across reservoirs and units.

*L. adecarboxylata* was found only in the environment, with 10 environmental isolates collected over 4 years (nine of which were *bla*_IMP-4_ positive). The three earliest isolates were collected on the same day in 2012 from the same shower (taps and different equipment), and are clustered on the Mash phylogeny (Figure 1), but had slight differences in insertion sequence and resistance gene profiles (Table S1). The remaining isolates were collected from drains between 2013 and 2015 in three different showers or bathrooms, mostly after cleaning,^39^ demonstrating the likely persistence of *bla*_IMP-4_, or its introduction to different strains of this species at these sites.

Nine *E. coli* clinical isolates were collected between 2007 and 2015: 3/9 harboured *bla*_IMP-4_ and these were genetically diverse (ST131, ST1674 and ST4995) consistent with multiple *bla*_IMP-4_ acquisition events. The six *K. oxytoca* isolates (five clinical and one environmental) were collected between 2010 and 2014. The clinical isolates were all collected in different wards of CRGH, including one collected from the burns unit. Two *bla*_IMP-4_-containing clinical isolates from June 2012 from different patients on two different wards [C39 (surgical) and C40 (medical)] were clonal (one SNV apart). This may represent transmission between patients before or after ward transfer or transmission via contaminated healthcare workers or equipment. Another two *bla*_IMP-4_-containing clinical isolates collected in 2010 and 2014 from two different wards were also related (C26 and C52; nine SNVs apart). Of note, the single environmental *K. oxytoca* collected from the burns unit was unrelated to all five clinical isolates (>27 000 SNVs apart). For *K. pneumoniae* and *S. marcescens*, host strains were genetically unrelated (>500 SNVs), consistent with new introduction of *bla*_IMP-4_-positive strains and/or *bla*_IMP-4_ horizontal gene transfer and acquisition by strains already circulating in the hospital.

### bla_IMP-4_-associated MGEs

**Figure 3.**
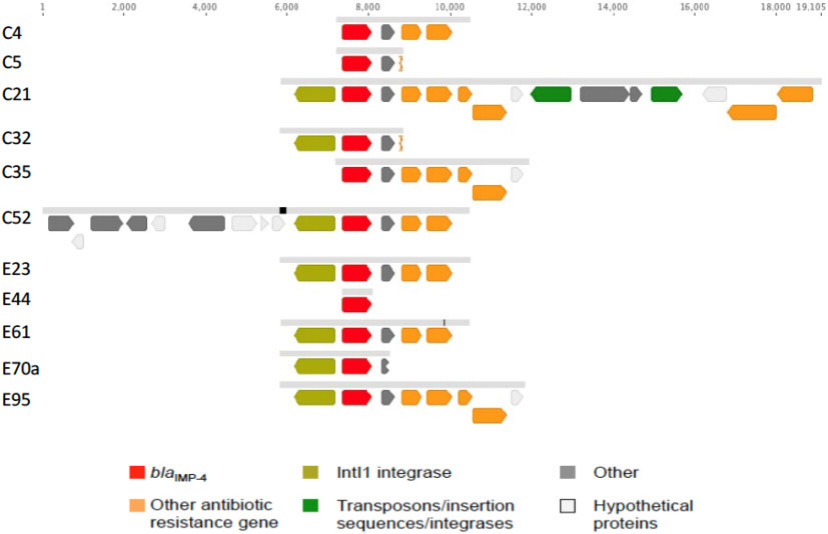
Alignment of 11 of the *bla*_IMP-4_-containing contigs from Illumina sequencing that represent all the IMP-4 contigs in this study. Pale grey bars between isolate sequences denote 100% sequence identity; thin black vertical lines in the bars are SNVs. Number of isolates with contigs identical to: C4, 23; C5, 9; C21, 3; C32, 1; C35, 1; C52, 1; E23, 67; E44, 1; E61, 1; E70a, 1; and E95, 5.

The *bla*_IMP-4_-containing contigs from Illumina sequencing ranged from 741 to 13 314 bp, with the majority (67/113) ∼4600 bp (Figure 3). These ∼4600 bp contigs were identical in all 67 isolates, except for one that contained an SNV in *catB3* (A>T). These 67 contigs, as well as 9 additional longer *bla*_IMP-4_-containing contigs contained several genes, including a transposase (Tn*3*), *bla*_IMP-4_, *qacG2* (quaternary ammonium resistance protein), *aacA4* (aminoglycoside acetyltransferase) and *catB3* (chloramphenicol acetyltransferase), consistent with the gene cassette previously described (*bla*_IMP-4_*-qacG2-aacA4-catB3*).^21^^,^^40^ Longer *bla*_IMP-4_-containing contigs (>13 000 bp) from three clinical isolates (*E. coli* ST131 and ST4995, and *S. marcesens*) collected between 2010 and 2013 were identical along their length, and also included the *ermE* gene (multidrug transporter) and the insertion sequences IS*116*, IS*110* and IS*902*, suggesting that this genetic motif has been shared between lineages and species, through either recombination or horizontal transfer. A further 25 isolates contained the *bla*_IMP-4_-*qacG2-aacA4-catB3* gene cassette, but without Tn*3*. In total, 101/113 (89%) of the *bla*_IMP-4_-containing isolates contained the *bla*_IMP-4_*-qacG2-aacA4-catB3* cassette array. The *bla*_IMP-4_-containing contigs from the remaining 11% of isolates were shorter; however, the regions flanking *bla*_IMP-4_ were identical to the other 89% of isolates, consistent with a common origin.

Based on plasmid typing from mapping short reads to reference plasmid sequences, 101/113 (89%) of the *bla*_IMP-4_-positive isolates also contained a common IncM2 plasmid (pEl1573-like), previously shown to carry *bla*_IMP-4_ in Australia.^40^^,^^41^ This plasmid was only found in isolates carrying *bla*_IMP-4_. Several other plasmids were potentially carrying *bla*_IMP-4_ in the remaining organisms, including pEC-IMP-like structures (*n *=* *19) (IncHI2A), p0801-IMP-like structures (*n *=* *7) and pIMP-HZ1-like structures (*n *=* *1) (IncN) (Table S1). Some organisms contained two plasmid types previously associated with *bla*_IMP-4_, namely pEl1573-like structures along with pEC-IMP-like structures (*n *=* *8), p0801-IMP-like structures (*n *=* *1) and pIMP-HZ1-like structures (*n *=* *1). In seven isolates (two *Acinetobacter baumannii*, two *C. freundii*, one *E. cloacae*, one *K. pneumoniae* and one *L. adecarboxylata*), no plasmid previously associated with *bla*_IMP_ was identified based on typing analysis from Illumina sequences. A total of 75/101 (74%) of the organisms carrying a pEl1573-like structure also contained other plasmid replicons of varying types (IncA/C, IncHI2A, ColRNAI, IncFII, IncHI2, IncFIB, IncN, IncR, IncFIA and IncHI1B), representing a diverse plasmid population within characterized *bla*_IMP-4_-positive isolates (Table S1).

**Figure 4.**
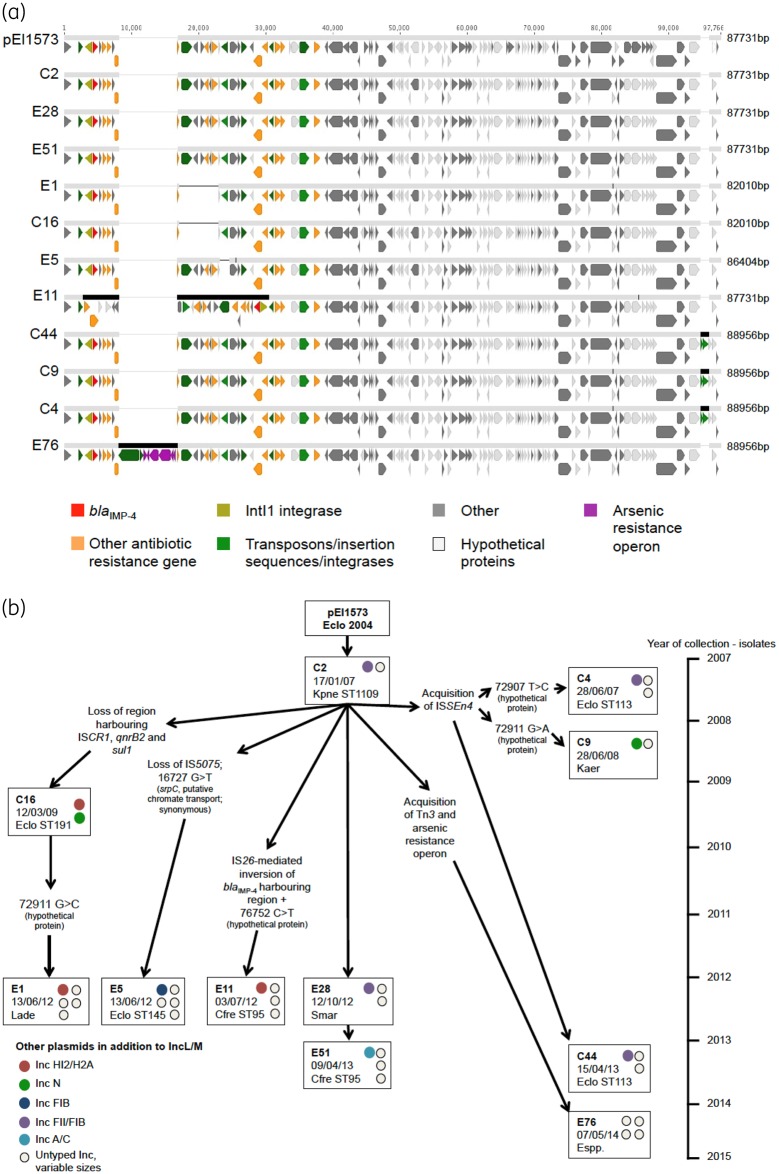
(a) Alignment of pEl1573 (GenBank accession number NC_019368.1) and the 11 closed IncM2 *bla*_IMP-4_-containing plasmid structures generated using MinION sequencing showing plasmid homology and rearrangement. Pale grey bars between isolate sequences denote 100% sequence identity; thin black vertical lines in the bars are SNVs. Thin/thick horizontal black lines denote gain/loss events of sequences with respect to each other. Individual plasmid sizes are denoted at the right-hand side of each sequence in the alignment. Isolate/species/date of isolation: C16, *E. cloacae*, 12 March 2009; E1, *L. adecarboxylata*, 13 June 2012; E5, *E. cloacae*, 13 June 2012; E28, *S. marcescens*, 12 October 2012; E51, *C. freundii*, 9 April 2013; C2, *K. pneumoniae*, 17 January 2007; C4, *E. cloacae*, 28 June 2007; C44, *E. cloacae*, 15 April 2013; C9, *K. aerogenes*, 28 June 2008; E76, *Enterobacter* spp., 7 May 2014; E11, *C. freundii*, 3 July 2012. (b) *bla*_IMP-4_ IncM2 plasmid evolution over 10 years showing putative horizontal transmission pathways amongst eight different species, and possible mutation and recombination/indel events. Both clinical and environmental isolates and multiple species are involved. The position of SNVs is with respect to the *bla*_IMP-4_ reference plasmid pEl1573 (GenBank accession number NC_019368.1).

ONT MinION long-read sequencing was performed on a random selection of 20 isolates that either: (i) appeared to contain the pEl1573 plasmid alone or with another *bla*_IMP-4_-associated plasmid; (ii) contained a non-pEl1573 plasmid likely to carry *bla*_IMP-4_; or (iii) did not appear to harbour any known *bla*_IMP-4_ plasmid based on plasmid typing of short reads. The *bla*_IMP-4_-containing contigs of these 20 isolates ranged from 59 247 to 360 711 bp in length (Table S2). Eleven had a pEl1573-like IncM2 plasmid containing *bla*_IMP-4_ (Figure 4a), consistent with importation of this structure into the hospital and its subsequent evolution (mutations, indel events and rearrangements) and interspecies transmission over 8 years (Figure 4b). Four isolates had *bla*_IMP-4_ on an IncFII plasmid (Figure S1, three species), one of several plasmid families associated with global transmission of antimicrobial resistance genes.^42^ Several mutations, indels and likely recombination events were observed within this plasmid backbone, with interspecies transfer over a 2 year period (2012–14). Two of these plasmids were found in clinical isolates (C45 and C50) of different species (*E. cloacae* and *C.* *freundii*, respectively) and were collected from the same ward 1 year apart, potentially showing ward-level persistence in an uncharacterized reservoir. The remaining isolates had *bla*_IMP-4_ located on an IncN3 plasmid (C52; 59 124 bp) or on plasmid structures without known Inc types [E61, 128 715 bp, closest BLASTn GenBank match MH909330.1 (60% identity); C54, 256 887 bp; and C47, 327 867 bp].

## Discussion

To our knowledge, this is the largest collection of *bla*_IMP-4_-positive isolates reported and characterized with WGS, and demonstrates a pattern of poly-species dissemination between patients and environmental reservoirs that is increasingly recognized for other transmissible carbapenemases such as *bla*_KPC._^43^^,^^44^ Almost half of the *bla*_IMP-4_-positive clinical isolates were collected from the burns unit, with evidence of extensive IncM2 plasmid/*bla*_IMP-4_ cassette transmission, and persistence of small identified clusters of strains. The remaining clinical isolates were found in several different medical and surgical wards, with healthcare-associated clonal transmission of some strains likely, alongside the introduction of new *bla*_IMP-4_-positive strains into the hospital and/or horizontal transfer of *bla*_IMP-4_-harbouring MGEs locally. Clinical isolates were most commonly found in wound swabs and mid-stream and catheter urine samples, probably in many cases representing colonization, and environmental isolates in wet sites such as shower and bathroom drains and sluices. *E. cloacae* was the predominant organism (as previously described in Australia)^22^ and had disseminated between patients and the environment, with evidence of prolonged transmission over 1–5 years for some clusters, suggesting a persistent environmental reservoir. *C. freundii* and *L. adecarboxylata* were predominant in the environment and seemed particularly resistant to both physical cleaning and chemical disinfectants,^39^ suggesting adaptive mechanisms for survival, and perhaps representing refractory reservoir species for the persistence of *bla*_IMP-4_ in these locations. The presence of *bla*_IMP-4_ in *E. coli* ST131 is of concern, as this lineage is associated with invasive extraintestinal infections.^45^

*bla*_IMP-4_ was detected most frequently in this study; however, three other carbapenemase genes were also found, consistent with recent evidence that other carbapenemases are also circulating in Australia.^46–48^ All *bla*_IMP-4_-positive isolates had five or more other resistance genes, resulting in MDR and facilitating selection under a range of different antimicrobial pressures. There was almost complete conservation of the *bla*_IMP-4_*-qacG2-aacA4-catB3* cassette array between all species described over the 10 year study period (with likely loss/gain of smaller MGEs, such as Tn*3* and some insertion sequences), showing the stability and promiscuity of this array between plasmids and species. IncM2 was the predominant *bla*_IMP-4_-carrying plasmid; however, several other Inc types were also found in association with *bla*_IMP-4_. Most isolates contained several different plasmids, allowing for recombination and the emergence of new vectors of *bla*_IMP-4_ such as IncF plasmids. Of particular note, IncF has not previously been widely described as carrying *bla*_IMP_ (seen only in one study to our knowledge),^49^ perhaps representing an emergent process of concern given the association of IncF plasmids with antimicrobial resistance genes, and highlighting the ongoing diversification of *bla*_IMP-4_ harbouring mobile plasmid vectors. Of 20 *bla*_IMP-4_ plasmids characterized by long-read sequencing, three (15%) were not associated with any known Inc type.

There are several limitations to our study. Whilst the clinical isolate collection was thought to be relatively complete, we did not perform routine screening for colonization with CROs throughout the hospital, and our sampling of the environment was more sporadic and largely focused on the burns unit in the context of an observed outbreak. In characterizing only single isolates per sample, we have probably underestimated the diversity of *bla*_IMP-4_-positive strains present in any given niche at any given timepoint, as demonstrated for other resistance genes in both patients and the environment.^43^^,^^50^ Even given these limitations however, our findings emphasize significant niche, species and *bla*_IMP-4_-associated MGE heterogeneity and therefore the considerable resources needed to investigate the full extent of the transmission networks involved in the dissemination of *bla*_IMP-4_. Mapping of short reads to reference plasmids has also been shown to have its limitations, in that it can misrepresent significant structural differences as similarities;^51^ however, we confirmed the genetic relatedness of a widely disseminated IncM2 *bla*_IMP-4_ plasmid with robust reconstruction of its structure from hybrid (short-/long-read) assemblies. Appropriate methods to quantify and characterize plasmid evolution and rates of interspecies transmission in highly diverse outbreaks are not yet available, and this meant that we were only able to perform a descriptive evaluation. Nevertheless, we have shown that inter-strain/species/plasmid transmission events appear common, and that rates of evolution and rearrangement for different plasmid types may be variable (e.g. IncF versus IncM2), with some plasmids diversifying more rapidly over time.

In summary, using high-resolution molecular typing with WGS, we highlight the diverse distribution of *bla*_IMP-4_ amongst a range of species and plasmids, and in both human and environmental reservoirs, in a single hospital setting in Australia over a decade. In several cases, the persistence of genetically indistinguishable strains in the environment was observed despite intensive decontamination measures. Surveillance and eradication in these contexts represent a considerable problem.

## Supplementary Material

dkz526_Supplementary_Data
