# Supplementary material for: Genomic dynamics of species and mobile genetic elements in a prolonged blaIMP-4-associated carbapenemase outbreak in an Australian hospital
Source: J Antimicrob Chemother. 2020 Jan 20;75(4):873–82. doi: 10.1093/jac/dkz526 (PMC7069471; doi:10.1093/jac/dkz526)
Supplement: dkz526_Supplementary_Data [file dkz526_supplementary_data.zip › Supplementary_data.docx]

**Supplementary data**

**Table S1.** Complete epidemiological, sequencing and typing data for each isolate (see separate Excel file).

**Table S2.** Structures and sizes of Unicycler assemblies (20 isolates) (see separate Excel file).


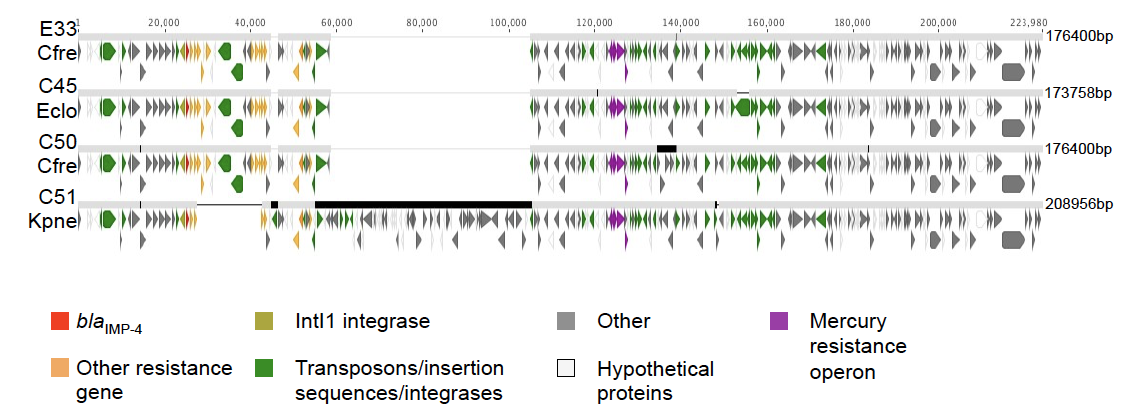


**Figure S1**. Alignment of the four IncF *bla*_IMP-4_ plasmid structures generated using MinION sequencing showing plasmid homology and rearrangement. Pale grey bars between isolate sequences denote 100% sequence identity; thin black vertical lines in the bars are SNVs. Thin/thick horizontal black lines denote gain/loss events of sequences respective to each other. Individual plasmid sizes denoted at the right-hand side of each sequence in the alignment.
